# A New Phylogenetic Framework for the Animal-Adapted *Mycobacterium tuberculosis* Complex

**DOI:** 10.3389/fmicb.2018.02820

**Published:** 2018-11-27

**Authors:** Daniela Brites, Chloé Loiseau, Fabrizio Menardo, Sonia Borrell, Maria Beatrice Boniotti, Robin Warren, Anzaan Dippenaar, Sven David Charles Parsons, Christian Beisel, Marcel A. Behr, Janet A. Fyfe, Mireia Coscolla, Sebastien Gagneux

**Affiliations:** ^1^Swiss Tropical and Public Health Institute, Basel, Switzerland; ^2^University of Basel, Basel, Switzerland; ^3^Istituto Zooprofilattico Sperimentale della Lombardia e dell’Emilia-Romagna: Centro Nazionale di Referenza per la Tubercolosi Bovina, Brescia, Italy; ^4^SAMRC Centre for TB Research, DST/NRF Centre of Excellence for Biomedical Tuberculosis Research, Division of Molecular Biology and Human Genetics, Faculty of Medicine and Health Sciences, Stellenbosch University, Stellenbosch, South Africa; ^5^Department of Biosystems Science and Engineering, ETH Zurich, Basel, Switzerland; ^6^McGill International TB Centre, Infectious Diseases and Immunity in Global Health, McGill University Health Centre and Research Institute, Montréal, QC, Canada; ^7^Mycobacterium Reference Laboratory, Victoria Infectious Diseases Reference Laboratory, Peter Doherty Institute, Melbourne, VIC, Australia; ^8^Institute for Integrative Systems Biology (I2SysBio), University of Valencia-CSIC, Valencia, Spain

**Keywords:** host–pathogen interactions, specificity, host range, genetic diversity, whole-genome sequencing

## Abstract

Tuberculosis (TB) affects humans and other animals and is caused by bacteria from the *Mycobacterium tuberculosis* complex (MTBC). Previous studies have shown that there are at least nine members of the MTBC infecting animals other than humans; these have also been referred to as ecotypes. However, the ecology and the evolution of these animal-adapted MTBC ecotypes are poorly understood. Here we screened 12,886 publicly available MTBC genomes and newly sequenced 17 animal-adapted MTBC strains, gathering a total of 529 genomes of animal-adapted MTBC strains. Phylogenomic and comparative analyses confirm that the animal-adapted MTBC members are paraphyletic with some members more closely related to the human-adapted *Mycobacterium africanum* Lineage 6 than to other animal-adapted strains. Furthermore, we identified four main animal-adapted MTBC clades that might correspond to four main host shifts; two of these clades are hypothesized to reflect independent cattle domestication events. Contrary to what would be expected from an obligate pathogen, MTBC nucleotide diversity was not positively correlated with host phylogenetic distances, suggesting that host tropism in the animal-adapted MTBC seems to be driven by contact rates and demographic aspects of the host population rather by than host relatedness. By combining phylogenomics with ecological data, we propose an evolutionary scenario in which the ancestor of Lineage 6 and all animal-adapted MTBC ecotypes was a generalist pathogen that subsequently adapted to different host species. This study provides a new phylogenetic framework to better understand the evolution of the different ecotypes of the MTBC and guide future work aimed at elucidating the molecular mechanisms underlying host range.

## Introduction

Tuberculosis (TB) remains a major concern both from a global health and economic point of view. With an estimated 10 million new human cases and 1.4 million fatalities in 2017, TB kills more people than any other infectious disease (World Health Organisation [WHO], 2018). Moreover, bovine TB is responsible for high economic losses in livestock production globally (Waters et al., 2012) and represents an ongoing threat for zoonotic TB in humans (Olea-Popelka et al., 2017). The causative agents of TB in humans and animals are a group of closely related acid-fast bacilli collectively known as the *Mycobacterium tuberculosis* complex (MTBC) (Brites and Gagneux, 2017; Malone and Gordon, 2017). The human-adapted MTBC comprises five main phylogenetic lineages generally referred to as *Mycobacterium tuberculosis* sensu stricto (i.e., MTBC lineages 1–4 and lineage 7) and two lineages traditionally known as *Mycobacterium africanum* (i.e., MTBC lineages 5 and 6) (de Jong et al., 2010; Brites and Gagneux, 2017; Yeboah-Manu et al., 2017). Among the animal-adapted members of the MTBC, some primarily infect wild mammal species (Malone and Gordon, 2017). These include *Mycobacterium microti* (a pathogen of voles) (Brodin et al., 2002), *Mycobacterium pinnipedii* (seals and sea lions) (Cousins et al., 2003), *Mycobacterium orygis* (antelopes) (van Ingen et al., 2012) and the “dassie bacillus” (rock hyrax) (Mostowy et al., 2004), which have been known for a long time, as well as the more recently discovered *Mycobacterium mungi* (mongooses) (Alexander et al., 2010), *Mycobacterium suricattae* (meerkats) (Parsons et al., 2013) and the “chimpanzee bacillus” (chimpanzees) (Coscolla et al., 2013). *Mycobacterium bovis* and *Mycobacterium caprae* on the other hand are mainly found in domesticated cattle and goats, but are also frequently isolated from several wild animal species which can act as reservoirs (Malone and Gordon, 2017). *Mycobacterium canettii* is also considered part of the MTBC based on nucleotide identity; however *M. canettii* is likely an environmental microbe only occasionally causing opportunistic infections in humans (Koeck et al., 2010; Supply et al., 2013). We therefore use the term “MTBC" to refer to all the above mentioned members except *M. canettii*. Many of the names of the animal-adapted MTBC species were originally coined based on the animal they were first isolated from. For example, *M. orygis* was first identified in a captive oryx (van Soolingen et al., 1994) but has since then been isolated from many different host species including humans (van Ingen et al., 2012). Thus, the actual host range of *M. orygis* remains ill-defined (Malone and Gordon, 2017). Similarly, for many of the animal-adapted members of the MTBC, only a few representatives have been isolated so far (e.g., only one in the case of the chimpanzee bacillus), limiting inferences with respect to the host range of these microbes. When studying host tropism, it is important to differentiate between maintenance hosts, in which the corresponding MTBC members traverse their full life cycle, including the transmission to secondary hosts, and spillover hosts, in which the infection leads to a dead end with no onward transmission (Malone and Gordon, 2017). For example, *M. tuberculosis* sensu stricto is well adapted to transmit from human to human (Brites and Gagneux, 2015) and is occasionally isolated from cattle or other animals which come in contact with humans (Ameni et al., 2011; Ghodbane and Drancourt, 2013). However *M. tuberculosis* sensu stricto is avirulent in cattle (Whelan et al., 2010; Villarreal-Ramos et al., 2018) and transmission from an animal back to humans is extremely rare (Murphree et al., 2011). Conversely, *M. bovis* is well adapted to transmit among cattle and does occasionally infect humans, mainly through the consumption of raw milk (Muller et al., 2013) or close contact with infected cattle, but transmission of *M. bovis* among immuno-competent humans is similarly uncommon (Blazquez et al., 1997). In contrast to *M. tuberculosis*, *M. bovis* has the ability to infect and maintain infectious cycles in other reservoir species such as badgers, red deers and possums (Delahay et al., 2001; Corner et al., 2012; Palmer et al., 2012).

The different members and phylogenetic lineages of the MTBC share a high nucleotide identity (>99.9%), and it has recently been suggested that they should be regarded as part of the same bacterial species (Riojas et al., 2018). The fact that these lineages also occupy different ecological niches, which is reflected in their host-specific tropism, supports a distinction into separate ecotypes (Smith et al., 2005). Yet, the host range of many of these animal-adapted MTBC members remain poorly defined, with respect to both maintenance and spillover hosts (Malone and Gordon, 2017). In this study, we present and discuss a new phylogenetic framework based on whole genome sequences covering all known MTBC ecotypes. Based on this novel framework, we challenge previous assumptions regarding the evolutionary history of the MTBC as a whole, and point to new research directions for uncovering the molecular basis of host tropism in one of the most important bacterial pathogens.

## Materials and Methods

### MTBC Genome Dataset

We downloaded 12,886 genomes previously published and accessible from the sequence read archive (SRA) repository by December 2017 as in (Menardo et al., 2018). To increase the representation of *M. bovis* we added to that dataset *M. bovis* genomes from different geographic locations (Trewby et al., 2016; Crispell et al., 2017; Malm et al., 2017) (Supplementary Table S1). After mapping and calling of variants (see below), phylogenetic SNPs as in Steiner et al. (2014) were used to classify genomes into human-adapted MTBC if they belonged to lineages 1–7 and if not, into non-human (hereafter referred to as “animal”) MTBC. All genomes determined as animal MTBC, as well as those classified as L5 or L6, were used for downstream analysis. We have furthermore newly sequenced four *M. orygis* genomes isolated in Australia in patients of South-Asia origin (Lavender et al., 2013), two dassie bacillus genomes isolated from two Hyrax imported from South-Africa to Canada (Cousins et al., 1994; Mostowy et al., 2004), eight *M. microti* isolated from wild-boar in Italy (Boniotti et al., 2014), two *M. bovis* strains isolated from patients in Switzerland and one *M. caprae* of unknown origin (Supplementary Table S1). For downstream analysis, we selected the genomes published in (Comas et al., 2013) as representatives of other human MTBC, giving a total 851 genomes used in this study (Supplementary Table S1). All isolates were handled in BSL3 facilities.

### Bacterial Culture, DNA Extraction and Whole-Genome Sequencing

The MTBC isolates were grown in 7H9-Tween 0.05% medium (BD) ±40 mM sodium pyruvate. We extracted genomic DNA after harvesting the bacterial cultures in the late exponential phase of growth using the CTAB method (Belisle and Sonnenberg, 1998). Sequencing libraries were prepared using NEXTERA XT DNA Preparation Kit (Illumina, San Diego, CA, United States). Multiplexed libraries were paired-end sequenced on an Illumina HiSeq2500 instrument (Illumina, San Diego, CA, United States) with 151 or 101 cycles at the Genomics Facility of the University of Basel. In the case of the *M. microti* isolates, DNA was obtained using the QIAamp DNA mini kit (Qiagen, Hilden, Germany). Libraries were also prepared with the NEXTERA XT DNA Preparation Kit and sequenced on an Illumina MiSeq using the Miseq Reagent Kit v2, 250-cycle paired-end run (Illumina, San Diego, CA, United States).

### Bioinformatics Analysis

#### Mapping and Variant Calling of Illumina Reads

The obtained FASTQ files were processed with Trimmomatic v 0.33 (SLIDINGWINDOW: 5:20) (Bolger et al., 2014) to clip Illumina adaptors and trim low quality reads. Reads shorter than 20 bp were excluded from the downstream analysis. Overlapping paired-end reads were merged with SeqPrep v 1.2 (overlap size = 15)^1^. We used BWA v0.7.13 (mem algorithm) (Li and Durbin, 2010) to align the reads to the reconstructed ancestral sequence of MTBC obtained as reported (Comas et al., 2010). There is no reconstruction available for an ancestral MTBC chromosome and thus the chromosome coordinates and the annotation used is that of H37Rv (NC_000962.3). Duplicated reads were marked by the Mark Duplicates module of Picard v 2.9.1^2^ and excluded. To avoid false positive calls, Pysam v 0.9.0^3^ was used to exclude reads with alignment score lower than (0.93^∗^read_length)-(read_length^∗^4^∗^0.07), corresponding to more than 7 miss-matches per 100 bp. SNPs were called with Samtools v 1.2 mpileup (Li, 2011) and VarScan v 2.4.1 (Koboldt et al., 2012) using the following thresholds: minimum mapping quality of 20, minimum base quality at a position of 20, minimum read depth at a position of 7x and without strand bias. Only SNPs considered to have reached fixation within an isolate were considered (at a within-isolate frequency of ≥90%). Conversely, when the SNP within-isolate frequency was ≤10%, the ancestor state was called. Mixed infections or contaminations were discarded by excluding genomes with more than 1000 variable positions with within-isolate frequencies between 90 and 10% and genomes for which the number of within-isolate SNPs was higher than the number of fixed SNPs. Additionally, we excluded genomes with average coverage lower than 15x (after all the referred filtering steps). All SNPs were annotated using snpEff v4.11 (Cingolani et al., 2012), in accordance with the *M. tuberculosis* H37Rv reference annotation (NC_000962.3). SNPs falling in regions such as PPE and PE-PGRS, phages, insertion sequences and in regions with at least 50 bp identities to other regions in the genome were excluded from the analysis (Stucki et al., 2016). SNPs known to confer drug resistance as used in Steiner et al. (2014) were also excluded from the analysis. For all animal MTBC genomes customized scripts in Python were used to calculate mean coverage per gene corrected by the size of the gene. Gene deletions with respect to the reference genome H37Rv were determined as regions with no coverage to the reference genome. We used those gene deletions to make correspondences with previously described regions of difference without identifying the exact limits of the different RD. To identify deletions of regions and genes absent from the chromosome of H37Rv (e.g., RD900), the unmapped reads resultant from the above described mapping procedure to H37Rv were obtained with Samtools v 1.2, mapped with reference to *M. canettii* (SRX002429) and annotated using as reference NC_015848, following the same steps described above (Supplementary Figure S1). We also recovered the unmapped reads from one representative of each human MTBC lineage and followed the same procedure (Supplementary Figure S1).

#### Phylogenetic Reconstruction

All 851 selected genomes were used to produce an alignment containing only polymorphic sites. This alignment was obtained using customized Python scripts and contained all polymorphic positions with no more than 50% of missing calls within the 851 genomes. The alignment was used to infer a Maximum likelihood phylogenetic tree using the MPI parallel version of RaxML (Stamatakis, 2006). The model GTR implemented in RAxML was used, and 1,000 rapid bootstrap inferences followed by a thorough maximum-likelihood search (Stamatakis, 2006) was performed in CIPRES (Miller et al., 2010). The best-scoring Maximum Likelihood topology is shown. The phylogeny was rooted using *M. canettii*. The topology was annotated using the package ggtree (Guangchuang et al., 2017) from R Core Team (2018)) and Adobe Illustrator CC. Taxa images were obtained from http://phylopic.org/. To remove redundancy and obtain a more even representation of the different MTBC groups for analysis of population structure and genetic diversity, we applied Treemer (Menardo et al., 2018) with the stop option *-RTL* 0.95, i.e., keeping 95% of the original tree length. The resulting reduced dataset was used for further analysis.

#### Population Structure and Genetic Diversity

Population structure was evaluated using Principal Component Analysis (PCA) based on SNP alignments using the R package *adegent* (Jombart, 2008). Genetic diversity was measured as raw pair-wise SNP differences within each MTBC lineage and ecotype if there were more than four genomes from a different geographic location, and as mean nucleotide diversity per site π using the R package *ape* (Paradis et al., 2004). π was calculated as the mean number of pair-wise mismatches among a set of sequences divided by the total length of queried genome in base pairs which comprise the total length of the genome after excluding repetitive regions (see above), equation 4.21 in Hartl and Clarck (2006). Confidence intervals for π were obtained by bootstrapping (1000 replicates) by re-sampling with replacement the nucleotide sites of the original alignments of polymorphic positions using the function *sample* in R Core Team (2018)). Lower and upper levels of confidence were obtained by calculating the 2.5th and the 97.5th quantiles of the π distribution obtained by bootstrapping (Nakagawa and Cuthill, 2007).

## Results and Discussion

### Genome-Based Phylogeny Reveals Multiple Animal-Adapted Clades

**FIGURE 1:**
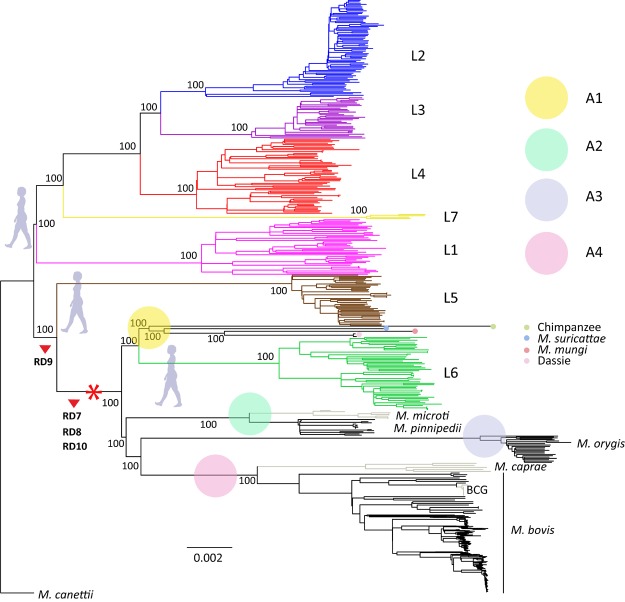
Maximum Likelihood topology of 322 human-adapted and 529 animal-adapted MTBC members. Branch lengths are proportional to nucleotide substitutions and the topology is rooted with *Mycobacterium canettii*. Support values correspond to bootstrap values. Main large deletions defining the animal-adapted MTBC are indicated by red arrow heads. The asterisk marks host range expansion within the MTBC.

We combined a total of 851 whole-genome sequences covering all known MTBC lineages and ecotypes. These included 834 genomes published previously, as well as four *M. orygis* genomes, two dassie bacillus genomes, eight *M. microti*, two *M. bovis* and one *M. caprae* newly sequenced here (Supplementary Table S1). We used a total of 56,195 variable single nucleotide positions extracted from these genome sequences to construct a phylogenetic tree rooted with *M. canettii*, the phylogenetically closest relative of the MTBC (Supply et al., 2013) (Figure 1). Our findings support the classification of the human-adapted MTBC into seven main phylogenetic lineages as previously reported (Gagneux et al., 2006; Gagneux and Small, 2007; Firdessa et al., 2013). Classical genotyping studies and genomic deletion analyses indicated a single monophyletic clade for all the animal-adapted MTBC defined by clade-specific deletions in the Regions of Difference (RD) 7, 8, 9 and 10 (Brosch et al., 2002; Mostowy et al., 2002), and our new genome-based analysis confirms that all known animal-adapted members of the MTBC share a common ancestor at the branching point which is characterized by these deletions. Of note, the human-adapted MTBC Lineage 6 also shares this common ancestor, which has led to the hypothesis that Lineage 6 might have an unknown animal reservoir (Smith et al., 2006); however, no such reservoir has yet been identified (Yeboah-Manu et al., 2017). Due to the limitations of standard genotyping (Comas et al., 2009) and the limited phylogenetic resolution of RDs in the MTBC (Hershberg et al., 2008), previous classifications have considered all animal-adapted ecotypes as part of one phylogenetic clade, recently referred to as MTBC “Lineage 8” (Gonzalo-Asensio et al., 2014). However, our new genome-based data revealed that these animal-adapted ecotypes form separate animal-adapted clades, some of which paraphyletic. For the purpose of this study, we discuss four of these animal-adapted clades which we named Clade A1 to A4.

### The Animal-Adapted MTBC Clade A1

**FIGURE 2:**
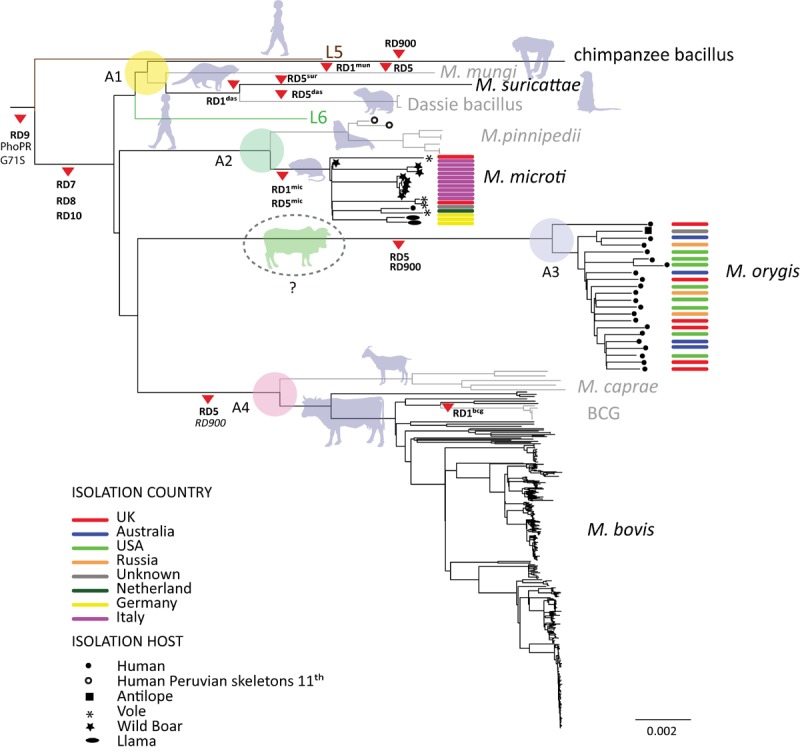
Topology showed in Figure 1 after collapsing all human-adapted branches. Branch lengths are proportional to nucleotide substitutions and the topology is rooted with *Mycobacterium canettii*. Support values are those of Figure 1. Main large deletions discussed in the text are indicated by red arrow heads and RD specific nomenclatures are indicated when available (Brodin et al., 2002; Mostowy et al., 2004; Alexander et al., 2010; Parsons et al., 2013; Dippenaar et al., 2015). Deletions which are polymorphic in terms of their presence or absence within main clades are indicated in italics.

One important finding from our phylogenomic analysis was that *M. mungi*, *M. suricattae*, the dassie bacillus and the chimpanzee bacillus form a separate Clade A1, which clusters with the human-adapted MTBC Lineage 6 (Figure 2). Based on limited previous genotyping data (Huard et al., 2006), it was hypothesized that the dassie bacillus shared a common ancestor with *M. africanum* (i.e., MTBC Lineage 6) (Huard et al., 2006; Brites and Gagneux, 2015). Our new whole genome data now confirms this hypothesis, and at the same time, highlight the fact that Clade A1 is more closely related to the human-adapted Lineage 6 of the MTBC than to the other animal-adapted ecotypes. This observation has important implications for our understanding of the original emergence of the animal-adapted strains and the evolutionary history of the MTBC as a whole as we shall discuss below. Regarding the animal-adapted MTBC, considering that Lineage 5 is human-adapted and basal to the RD7-10 defined lineages, the common ancestor defined by the deletions in RD7-10 could have been human-adapted pathogen as well (Brosch et al., 2002; Mostowy et al., 2002), and given that MTBC Lineage 6 is human-adapted (de Jong et al., 2010; Yeboah-Manu et al., 2017), the jump into animal hosts had to occur at least twice. Alternatively, the RD7-10 common ancestor might have been a generalist pathogen capable of infecting and causing disease in multiple host species (including humans), which was followed by a host-specialization of the different ecotypes and into humans during the emergence of Lineage 6.

Another important characteristic of clade A1 is the absence of the region encoded by RD1 in *M. mungi* (Alexander et al., 2010), *M. suricattae* (Parsons et al., 2013), the dassie bacillus (Mostowy et al., 2004) (Supplementary Table S2). RD1 encodes proteins that are essential virulence factors for MTBC in humans (further discussed below). Our data confirm that *M. mungi*, *M. suricattae*, the dassie bacillus all have deleted the region corresponding to RD1. This deletion is not present in the chimpanzee bacillus suggesting that RD1 might be essential for MTBC virulence in primates as proposed previously (Dippenaar et al., 2015).

### The Animal-Adapted MTBC Clade A2

Similar to Clade A1 that comprises pathogens adapted to wild animals, Clade A2 consists of two ecotypes mainly affecting wild animals, namely *M. microti* and *M. pinnipedii*. In addition, Clade A2 also includes MTBC genomes isolated from pre-Columbian human remains published previously (Bos et al., 2014). These ancient genomes are most closely related to *M. pinnipedii*, suggesting possible cases of zoonotic TB transmission resulting from the handling and consumption of seal or sea lion meat at the time (Bos et al., 2014). Contemporary *M. pinnipedii* is known to infect humans occasionally (e.g., zoo keepers or seal trainers), but no human-to-human transmission has been documented to date. *M. microti* was originally isolated from voles in the 1930s (Wells, 1937), but has since then been found in cats, pigs, llamas and immune-compromised humans (Brodin et al., 2002; Frota et al., 2004; Smith et al., 2009a,b). Here we report 8 new *M. microti* genomes isolated from wild boar. Based on the 15 *M. microti* genomes included in this analysis, some host-specificity of particular sub-groups with this ecotype might be suggested, but analysis of a larger sample is needed to explore this possibility further. To our knowledge, *M. microti* has not been reported outside Europe, as infections in llamas pertain to captive animals in Europe (Oevermann et al., 2004) and represent probable spillovers from other hosts. Furthermore, the *M. microti*–like strain isolated from a rock hyrax has been likely misclassified (Clarke et al., 2016). Many of the animals species infected by *M. microti* occur across Eurasia which might therefore also correspond to the geographic range of *M. microti*. One of the important characteristics of all *M. microti* strains is the deletion of RD1 (RD1^mic^) (Brodin et al., 2002), which is independent of the one described for some of the members of Clade A1, and which is the most important virulence attenuating mutation in the *M. bovis* BCG vaccine (RD1^BCG^) (Pym et al., 2002). In support of the low virulence of *M. microti* in humans, and in contrast to *M. bovis* and *M. orygis* (see below), we detected only one infection with *M. microti* (from an immune-compromised patient (van Soolingen et al., 1998) among all the human isolates queried in the public domain (see Materials and Methods).

### The Animal-Adapted MTBC Clade A3

**FIGURE 3:**
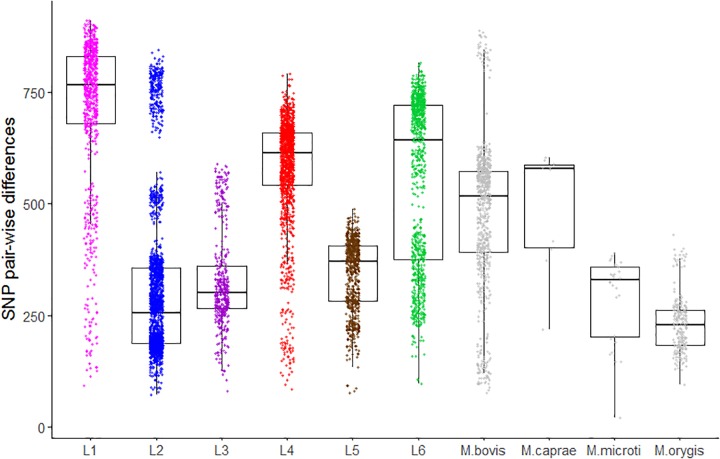
Pair-wise SNP distances within lineage and ecotype from human and animal-adapted MTBC, respectively. Each box corresponds to the 25 and 75% quantiles, the black line represents the median and the whiskers extend to 1.5 times the interquartile range.

In contrast to the animal Clades A1 and A2 that include multiple MTBC ecotypes infecting various wild animal host species, A3 comprises only genomes belonging to *M. orygis*. Even though *M. orygis* has been isolated from many different wild and domestic animals (Dawson et al., 2012; Gey van Pittius et al., 2012; van Ingen et al., 2012; Thapa et al., 2015, 2016; Rahim et al., 2017), a large proportion of isolates reported to date are actually from human TB patients. One of the first detailed studies reporting on the genotypic properties of *M. orygis* strains included a total of 22 isolates, 11 of which originated from humans (van Ingen et al., 2012). The majority of the remaining isolates came from various zoo animals from the Netherlands and South Africa, which included three waterbucks, two antelopes, one deer and one oryx. A recent study from New York reported whole genome data from eight *M. orygis* isolates from human patients (Marcos et al., 2017). Another recent report from Birmingham, United Kingdom identified 24 *M. orygis* among 3,128 routinely collected human MTBC isolates (Lipworth et al., 2017). Similarly, eight *M. orygis* isolates were reported among 1,763 human TB cases from Victoria, Australia (Lavender et al., 2013), the genomes of four of which are newly reported here (Figures 1, 2). Importantly, all human *M. orygis* isolates, were from patients born in India, Pakistan, Nepal or “South Asia,” except for one with a reported origin in “South East Asia” (Dawson et al., 2012; van Ingen et al., 2012; Lavender et al., 2013; Marcos et al., 2017). This also includes one patient who immigrated from India to New Zealand and infected a dairy cow there (Dawson et al., 2012). One recent study reported 18 *M. orygis* isolates from dairy cattle in Bangladesh (Rahim et al., 2017). These isolates grouped into three distinct MIRU-VNTR clusters, with the largest cluster including two additional *M. orygis* isolates from captive monkeys. The authors propose that *M. orygis* is endemic among wild and domestic animals across South Asia and thus of relevant One Health significance. Based on the available evidence summarized above, and given that *M. orygis* shares its most recent ancestor with Clade 4 (Figure 1), which comprises *M. bovis* and *M. caprae*, whose evolutionary success is mostly due to the ability of effectively infecting domestic animals (further discussed below), we extend this notion, and hypothesize that *M. orygis* is primarily a pathogen of cattle in South Asia, leading to zoonotic TB in humans through e.g., the consumption of raw milk. This hypothetical scenario offers a parsimonious explanation for why *M. orygis* has repeatedly been isolated from South Asian migrants living in low TB-endemic countries in Europe, United States, and Australia (Dawson et al., 2012; van Ingen et al., 2012; Lavender et al., 2013; Marcos et al., 2017). The genetic distance among the *M. orygis* identified in this study is compatible with such scenario, as the genomes of these isolates differ on average by 231 SNPs, supporting independent infections in the patients’ countries of origin (Figure 3). Broader in-depth molecular analyses of cattle TB in South Asia, for which little data currently exist despite it representing a major public health threat (Rahim et al., 2017; Srinivasan et al., 2018) are needed to verify our hypothesis. Regarding *M. orygis* reported in animals other than cattle, our hypothesis would suggest that these likely represent spillovers or reservoirs from infected cattle, similar to the situation seen in *M. bovis* (Malone and Gordon, 2017). In support of this view, except for one case isolated from a free-ranging rhinoceros in Nepal (Thapa et al., 2016), all *M. orygis* reported in un-domesticated animals were associated with zoos, farms or other forms of captivity where these wild animals might have come into contact with *M. orygis* infected cattle or humans (Gey van Pittius et al., 2012; van Ingen et al., 2012; Thapa et al., 2015; Rahim et al., 2017).

### The Animal-Adapted MTBC Clade A4

Clade A4 includes the classical members of the animal-adapted MTBC, i.e., *M. bovis*, *M. caprae* and all the *M. bovis* BCG vaccine strains (Figure 2). Much work has been done on the genetic characterization of these MTBC members (Mostowy et al., 2005; Huard et al., 2006; Smith et al., 2006; Muller et al., 2009; Copin et al., 2014; Malone and Gordon, 2017), and thus we will not discuss these in any further details here. One exception is the deletion RD900, which has been described as a region specific to L6 and for which, presence and absence in *M. bovis* has been disputed (Bentley et al., 2012; Malone et al., 2017). The results of mapping with respect to *M. canettii* reads which remained unmapped to the chromosome of H37Rv revealed that RD900 is polymorphic within *M. bovis*, within BCG strains and within *M. caprae*. In contrast, the region encoded by RD900 was deleted in all *M. orygis* genomes analyzed (Figure 2).

We end this section by speculating that if our hypothesis regarding the host range of *M. orygis* is true, Clade A3 and Clade A4 might reflect the two independent cattle domestication events known to have occurred in the Fertile Crescent and Indus Valley, respectively (Loftus et al., 1994). The corresponding domesticated forms emerging form the ancestral aurochs (*Bos primigenius*) are the sub-species *Bos taurus* and *Bos indicus*. Hence, *M. bovis* might have adapted to *B. taurus* whereas *M. orygis* might be better adapted to *B. indicus*. While highly speculative at this stage, this hypothesis could be tested experimentally for instance by comparing the virulence of *M. bovis* and *M. orygis* in macrophages from *B. taurus* or *B. indicus* (Villarreal-Ramos et al., 2018).

### MTBC Genetic Diversity and Host Specificity

**FIGURE 4:**
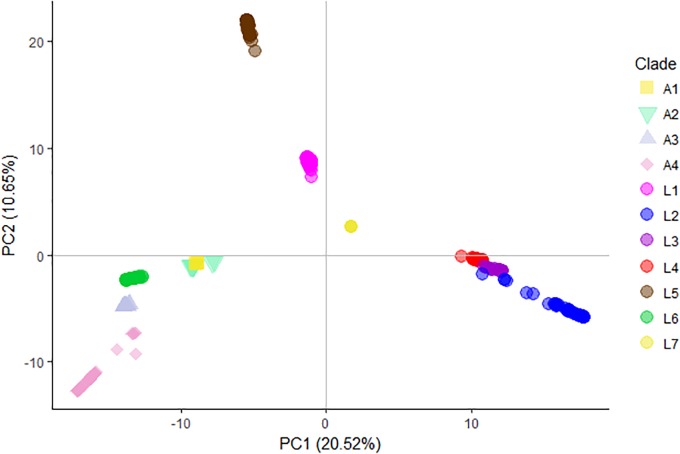
Principal component analysis (PCA) derived from whole-genome SNPs. The two first principal components are shown.

From an ecological perspective, pathogen diversity is generally positively correlated with host diversity especially in the case of obligate pathogens (Kamiya et al., 2014). Given the broad MTBC range of hosts, we explored how the genetic diversity was partitioned within the MTBC and if the genetic diversity of the animal-adapted MTBC was higher than that of the human-adapted MTBC. To obtain a more balanced representation of the different MTBC groups and remove redundancy caused by an over-representation of very closely related isolates which tell us little about macro-evolutionary processes, we used Treemer (Menardo et al., 2018) and reduced our dataset from 851 to 367 genomes while keeping 95% of the original total tree length. We performed principal component analysis (PCA) on the matrix of SNP distances correspondent to the non-redundant data set (*n* = 367) (Figure 4). The resultant groups correspond largely to the results obtained with the phylogenetic approach. The first principal component (PC1) explained 20.5% of the variation in genetic differences and highlights the contrast between “modern” human MTBC lineages (Lineages 2, 3 and 4) and Lineages 1, 5 and 7, which on their own formed very distinct groups. Lineage 6 appeared closer to the animal MTBC but separated from clade A1. Interestingly, despite a clear separation between the human-adapted and animal-adapted MTBC (except for Lineage 6), PC1 contrasted more prominently the different human-adapted lineages than the different animal-adapted ecotypes (Figure 4).

**FIGURE 5:**
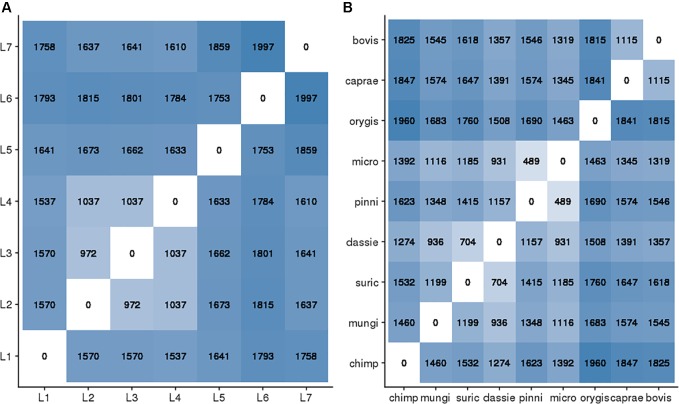
Pair-wise SNP distances between one randomly chosen representative of each human adapted MTBC lineage **(A)** and animal-adapted MTBC **(B)**. Darker and lighter blue indicate higher and lower genetic distances respectively.

As a measure of genetic diversity, we estimated the mean nucleotide diversity per site (π) of human versus animals isolates. The estimates indicated that two randomly picked human isolates differed on average by 0.0345% nucleotide differences (95% CI: 0.0337–0.0352%) whereas animal isolates differed on average by 0.0313% (95% CI: 0.0305–0.0321%). Despite non-overlapping confidence intervals, the difference between our π estimates was small (0.003%). A higher genetic diversity of human-adapted MTBC relative to animal-adapted MTBC was also uncovered by (Zimpel et al., 2017) using other estimators. The estimates of π reflect both the diversity within each lineage/ecotype, as well as the diversity between lineages/ecotypes, resulting from older evolutionary events leading to the emergence of the latter. Whereas our sampling of the human MTBC reflects both pre- and post-lineage diversification reasonably well, the animal MTBC samples are most likely a poor representation of the genetic diversity resulting from diversification processes within each ecotype, with the possible exception of *M. bovis* (Figure 3). We thus compared the raw SNP differences among one random representative of each human and animal-adapted MTBC lineage and ecotype (Figure 5). The SNP differences accumulated in the different human-adapted lineages can be as high, or even higher than the genetic differences that separate MTBC strains infecting a broad taxonomic range of mammal species other than humans. Thus host-specificity in the MTBC cannot be easily explained by quantitative genetic differences among the different animal-adapted MTBC ecotypes.

In the light of the fact that in the MTBC, as in other bacteria, genomic variants caused by large deletions are pervasive (Bolotin and Hershberg, 2015) and genomes evolve toward a reduction of gene content as no horizontal gene transfer has been found in extant populations of the MTBC, it is also unlikely that the acquisition of new genes underlies host specificity. In support of this, after mapping reads using *M. canettii* as a reference, we found no regions that would be present in all representatives of each of the different animal ecotype genomes and absent from human-adapted MTBC genomes. Several genomic deletions have been described in the genomes of animal-adapted MTBC members which we could confirm here (Supplementary Table S2). Some of those deletions, e.g., RD1 and RD5, have been shown to impact virulence in different ways (Lewis et al., 2003; Dippenaar et al., 2015; Ates et al., 2018b). In the case of RD1 and RD5, the deletion events seem to have occurred independently in different animal MTBC ecotypes (Figure 2) suggesting that the former have provided a fitness gain and were involved in the adaptation to new hosts (Brodin et al., 2002; Dippenaar et al., 2015; Ates et al., 2018b). However, RD5 has also independently evolved and shown to impact virulence in the human adapted L2 Beijing sub-lineage (Ates et al., 2018a). Taken together, this suggests that MTBC genomes are extremely robust in terms of host adaptation, and that interactions between different genes in the different ecotypes could be key determinants of host specificity in the MTBC as suggested, e.g., by the results of (Gonzalo-Asensio et al., 2014; Ates et al., 2018b).

### Evolutionary Scenarios for the Evolution of the Animal-Adapted MTBC

The different MTBC members have adapted to infect a broad range of mammalian species, ranging from micro-mammals with short life-spans to humans, indicating that host shifts to distantly related hosts have occurred throughout the evolution of the MTBC. However, these host shifts have not emerged from any random phylogenetic branch of the MTBC as most of the human-adapted MTBC lineages are monophyletic and possibly locally adapted to different human populations (Fenner et al., 2013; Gagneux, 2018). Host range expansion seems to have occurred after the split between Lineage 5 and the ancestor of Lineage 6 and all the animal ecotypes (Figure 1). One plausible scenario as mentioned in the discussion of clade A1 is that the ancestor pathogen of the extant animal-adapted MTBC and Lineage 6 was a generalist with the ability to cause infections in many different kinds of hosts. A series of genetic events have been put forward by Gonzalo-Asensio et al. (2014) to explain the decreased virulence of *M. africanum* L5 and L6 and the animal MTBC members compared to *M. tuberculosis* sensu stricto. A non-synonymous mutation on the codon 71 in the *phoR* gene (Figure 2) which has emerged in the common ancestor of *M. africanum* L5 and L6 and of the animal-adapted strains, if transferred to a *M. tuberculosis* sensu stricto background leads to decreased virulence in mice and primary macrophages (Gonzalo-Asensio et al., 2014). This decrease in virulence is mediated by a decrease in the secretion of ESAT-6 which among other virulence factors is regulated by *phoPR* genes. The work of Gonzalo-Asensio et al. (2014) shows that in L6, the loss of virulence was compensated by the RD8 deletion which restored the secretion of ESAT-6 independently of *PhoPR*. RD8 is common to L6 and all the animal ecotypes (but not L5, Figure 2), thus how the effects of *PhoPR* are restored in L5 remains unknown. This and related events could be at the origin of a putative generalist pathogen with compromised virulence in its original human host, and for which infecting other hosts represented fitness gains leading to the host range expansion we see today.

**FIGURE 6:**
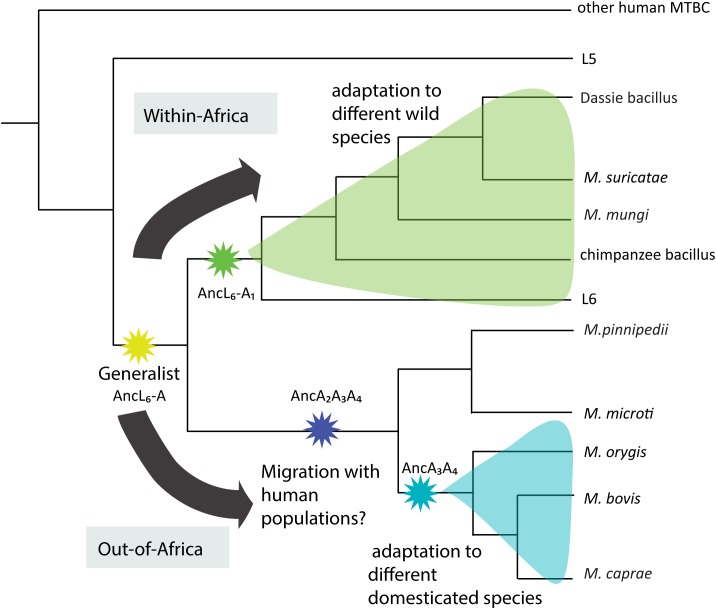
Schematic illustration of the putative evolutionary history of the animal-adapted MTBC. The length of the branch is not proportional to genetic distances.

Based on the known geographic ranges of the animal-adapted MTBC ecotypes, we hypothesize two main divisions after the emergence of the ancestor of L6 and the animal ecotypes (Anc_L6–A_, Figure 6); A series of specialization events which have occurred within Africa leading to the emergence of L6 in humans and clade A1 in several wild mammal species. With the exception of the chimpanzee bacillus, these ecotypes have all been sampled in Southern Africa (Clarke et al., 2016). However, the extant geographic distributions of the hosts are not restricted to Southern Africa (except for Meerkats), additionally they have several overlapping areas and as a whole, form a continuum ranging from West-Africa to Southern-Africa^4^. Another series of specialization events might have happened outside Africa as suggested by the extant distribution of *M. orygis* and *M. microti* (Figure 6). Given that the maintenance hosts of strains that comprise A3 and A4 are domesticated species, one possible scenario is that the ancestor of Anc_A2–A3–A4_ was carried by human populations as they migrated from Africa to the rest of the world (Figure 6). This ancestor could have been transferred posteriorly to different cattle and other livestock species which were domesticated independently outside Africa in different parts of world as suggested in the discussion of clade A3 above, and become extinct in human populations. The example of the three human Peruvian remains circa 1000 years old, which were infected with what is known today as *M. pinnipedii* (Bos et al., 2014) support the plausibility of such a scenario. Alternatively, Anc_A2-A3-A4_ might have been brought outside Africa by another migratory species with close contact to livestock. The jump from the ancestor Anc_A2-A3-A4_ to clade A2, which comprises such different host species, is not easily explained without invoking an environmental reservoir. This cannot be excluded as *M. bovis* and *M. microti* can possibly survive in the environment (Courtenay et al., 2006; Kipar et al., 2014).

The biology of pathogen jumps into new hosts involves three main steps (Woolhouse et al., 2005); (i) exposure of the pathogen to a new environment, i.e., contact rates between hosts or between hosts and an environmental reservoir, (ii) the ability to infect the new host, which most commonly decreases with the genetic distance from the ancestral host, and (iii) transmissibility within the new host population. Generally, when the complete host ranges and the known geographic distributions are taken into account in the animal-adapted MTBC ecotypes, geographic proximity between hosts and therefore contact rates seemed to have played a more important role in determining host range and specialization than genetic distances among hosts. A corollary of these considerations, and given that one important contributor to (ii) is the ability to avoid or supress the host immune system, is that the immune repertoire of the host may have played a less important role in determining the host range of the different animal MTBC ecotypes compared to (i) or (iii) as long as the hosts were mammalian species. There are exceptions to this, e.g., within Clade A1, moongoses and meerkats belong to the same taxonomic family (Clarke et al., 2016). However, in this case, host geographic range, ecology and genetic distances are not independent, blurring conclusions. One important characteristic common to all host species in which the different MTBC members cause sustainable infections is that they attain high population densities, even if predominantly seasonally as in the case of pinnipeds (Cassini, 1999). This characteristic might have been one of the most important determinants in the evolution of the different MTBC ecotypes and in particular, of their mode of transmission. Whereas the ability to cause pulmonary infections is essential for transmission among humans, in other animals, routes of infection other than aerosol transmission seem to play an important role, e.g., grazing contaminated pasture leads probably to a significant proportion of infections by *M. bovis* in cattle (Phillips et al., 2003), *M. mungi* can transmit directly through abrasions resultant from foraging activity of banded mongoose (Alexander et al., 2010; Malone and Gordon, 2017), and transmission through skin lesions in *M. microti* has also been suggested (Kipar et al., 2014).

## Conclusion

There are several reports about animal-adapted members of the MTBC infecting humans, wild and domestic animals, but an overarching analysis of all information available is required. In this study, we have combined all available information about animal-adapted MTBC strains and expanded it by sequencing more animal-adapted MTBC strains gathering the most comprehensive whole genome dataset of animal-adapted MTBC to date. We have used genomic analysis to elucidate the evolutionary history of the animal-adapted MTBC and have confirmed that the former are paraphyletic and that at least four different main clades can be defined. The phylogeny presented together with the known host range would be compatible with two scenarios during the evolutionary history of the non-human MTBC, both involving more than one host jump. One scenario would present the ancestor of the group including L6 and all animal-adapted clades as a generalist capable of infecting a wide group of mammals, and different host adaptations would have occurred thereafter. An alternative scenario proposes that the ancestor of L6 and animal-adapted MTBC was adapted to humans, and subsequent host jumps lead to the host specificity of the four clades.

We found no correlation between genetic diversity of the pathogen and the phylogenetic distance of the host, as animal-adapted MTBC strains are not more diverse in average than human-adapted strains. Based on the current known host-ranges and geography of the animal-adapted MTBC, we propose that host expansion has been driven to a great extent by host geographical proximity, i.e., by contact rates among different species of mammals, and by high host population densities rather than by host genetic relatedness.

## Author Contributions

DB, CL, MC, and FM have analyzed the data. DB, SG, and MC wrote the manuscript. MBB, RW, AD, SP, MB, CB, SB, and JF contributed reagents and performed the experiments.

## Conflict of Interest Statement

The authors declare that the research was conducted in the absence of any commercial or financial relationships that could be construed as a potential conflict of interest.

## Footnotes

1 https://github.com/jstjohn/SeqPrep

2 https://github.com/broadinstitute/picard

3 https://github.com/pysam-developers/pysam

4 http://www.iucnredlist.org/

## Supplementary Material

FIGURE S1 Schematic view of the bioinformatic procedure to characterize the identity of reads not mapped with respect to the H37Rv chromosome.

TABLE S1 Final set of genomes used in the analysis and some metadata associated. NA stands for non-available.

TABLE S2 Description of gene deletions found to be common to all representatives of each of the main Clades discussed in the text and their corresponding regions of difference.
