# Supplementary figures and images for: A New Phylogenetic Framework for the Animal-Adapted Mycobacterium tuberculosis Complex
Source: Front Microbiol. 2018 Nov 27;9:2820. doi: 10.3389/fmicb.2018.02820 (PMC6277475; doi:10.3389/fmicb.2018.02820)

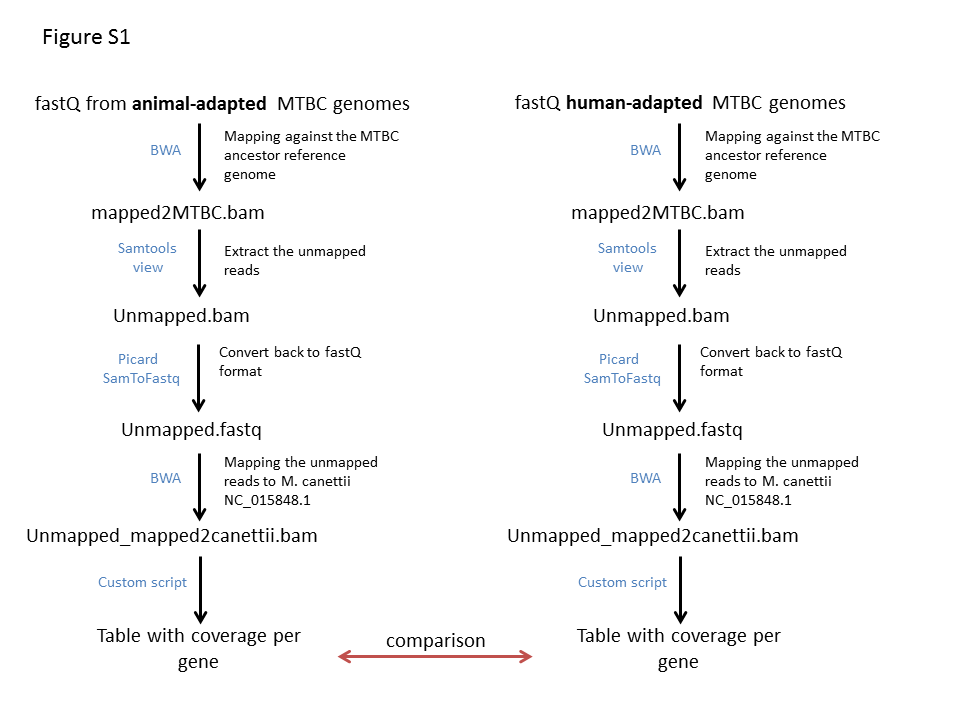

Supplement: FIGURE S1 — Schematic view of the bioinformatic procedure to characterize the identity of reads not mapped with respect to the H37Rv chromosome. [file Image_1.tif]
